# Supplementary material for: Patterns of Intron Gain and Loss in Fungi
Source: PLoS Biol. 2004 Nov 30;2(12):e422. doi: 10.1371/journal.pbio.0020422 (PMC532390; doi:10.1371/journal.pbio.0020422)
Supplement: Table S1 — Also available at http://genes.mit.edu/NielsenEtAl/. (4.3 MB ZIP). [file pbio.0020422.st001.zip › NielsenEtAl/html/407.html]

AN2494.1.NCU04642.1.MG00770.1.FG10205.1


```
 CLUSTAL W (1.82) Multiple Sequence Alignments - Introns Inserted


Sequence 1: NCU04642.1	591 aa
Sequence 2: MG00770.1	582 aa
Sequence 3: FG10205.1	583 aa
Sequence 4: AN2494.1	562 aa
Alignment Length: 621 aa
Number Identitical Residues: 189 aa
Alignment Score (without introns) 11813


MG00770.1 	-MPALSDEEWAEIAG-EIPQASDPFIQKYLQGRDELVSQEQRQRSDHAFKQSLSPIARRA
NCU04642.1	MTNQITDEEWADILAHELPTQNDPILQKYHAARAALIAEEQKQRSDHVFRQSLSPLARRA
FG10205.1 	----MSNEEWDEVAQ-DIPSLSDPFLQQYLTGRANLISQEKKSRSDASFRASLSPIAQRA
AN2494.1  	--------------------MAQGIIENHLSKRSALLEQEKTSRHDYAFKQSMSPVAKTA
          	                      : :::::   *  *: :*: .* *  *: *:**:*: *

MG00770.1 	CAIVERIRLEENGSVWTTALEEKVAERG-----HVFPGMMFSLAKDAMETTQLWRIVRRM
NCU04642.1	CSIVSRIRLEEQKAIWTPDLEEQLARKEENNGIIIHPGMMFTLAKETMEATKLWRIVRKM
FG10205.1 	SDIVDRIRDQENDTVWTPQVEEDLAQAG---NECIFPGMMFMLAKDRMEKTNLWNIVRRM
AN2494.1  	AKVLADIRKKELKSIWSPTTTSNEKRDLK---DVTFPGMSFTLSRDRMQKTALWKIVSAM
          	. ::  ** :*  ::*:.   ..  .  .      .*** * *::: *: * **.**  *

MG00770.1 	PKGALLHGHNDAMADFDFLIAVLLDTEGMCVACGE-GGLATAQQ-REERIPEFRFVKSPP
NCU04642.1	PKGALLHSHCDAMVDFDFLLGVVLETEGMGIATPERGGLGTEER-RREAEVVVKFVGELE
FG10205.1 	PKGALLHAHMDAMVNFDFLFDQLVKMPGMHISSDR--PLNTDES-REDATPNFRYRKQAD
AN2494.1  	PKGCLLHAHMEAMIDIDWMIEQAIELPGYYISSPVPLCPRTGSAGKWSEPFQFSYRPRLG
          	***.***.* :** ::*:::   :.  *  ::        * . .: .    . :     

MG00770.1 	ADVDAA-------SLWRDD-------YKAGTFVPITKAADSFPSSSSSSSDGSGGGRAAF
NCU04642.1	RKVDAK-------SIWAGDEEGGGGGYEPGTFINLVEAADAFPD----------GGRKGF
FG10205.1 	AEA----------SIWDES-------YKAGTFVPLIKAADEFLH----------GGRAGF
AN2494.1  	SGANARNGSEPEISIWQAG-------YTPGRLVPVAAAAKMYPG-----------GVEAF
          	  ..: ..:... *:*  .       * .* :: :  **. :             *  .*

MG00770.1 	TAWLKSRVTLSRTDSTEQHHGQDHIWRKFINCFRITGSMMHYEPVFRRFLQRVMTLLHED
NCU04642.1	LQWLKSRCILSQTDAIEQRHGSAEIWRKFIRCFGVIGSMLHYEPIWRRFLRRLLQQLVED
FG10205.1 	FKWLKGRCTLSVTDSHEQHHGIDAIWVKFAKCFMVCATIIHYEPMLRVFLRELMKNLKDD
AN2494.1  	KAWAVSRMTINEEEALEHHHGIDDVWDKFRSCFLAIGGLFFTEPIFRRCIPRLLQQLHDD
          	  *  .*  :.  :: *::**   :* **  **   . ::. **: *  : .::  * :*

MG00770.1 	GVAWAELR~HTWPLDYRREGREEPETDYMHMFTVIDEEVTAFKQANPG--FWGMRMIWSS
NCU04642.1	GVYWVEVR2FAWQLDYCREGCTTPEPDYTAMFNVIEEEVAAFKATPEGSSFWGLRMIWTT
FG10205.1 	GVNWAELR2MTWSLNYCRDQQEEPEKDYNHLFEVIREEIDNFKKSPEGEGFWGLTTIWTS
AN2494.1  	GIKYVELR~LAPNP-FYREGSNEAEPDFLYFLQCFQEEVDAYMSSPTGRGFWGAKIIWTA
          	*: :.*:*  :    : *:    .* *:  ::  : **:  :  :  * .***   **::

MG00770.1 	LRSADERTMASDMMLCVETKLAFPHLVVGYDVVGQEDAGPSLKDLLPRFAWFKKLCAQEG
NCU04642.1	VRAFGPRTIIQSMNDCIATKINFPHLIAGYDLVGPEDAGRHLTDLLPELFWFRKQCAAEG
FG10205.1 	LRSWPTRTIVENMDFCIATKMAFPDLIAGYDLVGPEDMGRPLSDLLPELFWFRKQCAVEN
AN2494.1  	IRSFPDDLIKESMIHCLNSKKAYPSVITGFDFVGQEDAGRPLVDLLPLCKWFQQQCADEQ
          	:*:     : ..*  *: :*  :* ::.*:*.** ** *  * ****   **:: ** * 

MG00770.1 	VNIPFFFHAGETLGDGTETDQNLFDAVLLGTRRIGHGFSLHKHPLLIDLVKQKRILIESC
NCU04642.1	VEIPFFLHAGETLGDGDAVDHNLFDALLLGARRIGHGFSLYKHPQLIKAVKDKRVLIESC
FG10205.1 	VNLPFFFHAGETLGDGTDTDSNIFDAILLGTRRIGHGFSLFKHPLLIDMVKEKRILIESC
AN2494.1  	LQIPFFLHAGECLGDGNDTDSNLVDAILLNSRRIGHAFSLYKHPLLIDLVKDKNILIEMC
          	:::***:**** ****  .* *:.**:**.:*****.***.*** **. **:*.:*** *

MG00770.1 	PISNEVLRLCGSILSH2PLPALLARGVPCALSNDDPGMLGQDTAGATHDFWQALQGWDNL
NCU04642.1	PISNEVLRLTGSIMQH~PLPALLARGVPCALCNDDPAILGQDMAGMTHDFWQALQGWENL
FG10205.1 	PISNEVLRLCGSVTAH~PLPALLARGVACSLCNDDPAMLGQDTAGMSHDFFQAVQGWKNL
AN2494.1  	PISHEVLRLTSNILMH~PMPALQARGVAVSLNNDDPAVLGHGKNGLSHDFYQVTAAFENT
          	***:***** ..:  * *:*** ****. :* ****.:**:.  * :***:*.  .:.* 

MG00770.1 	GLGGLGSLAENSIRWSAFEDETDAQWKSSIQQASLGSGIKAQRLKEWAIKWEEFCLWIVD
NCU04642.1	GLAGLGSLAENSVRWAAFEDQTADEWARDVREASMGSGTKAERLKQWAVEWEKFCVWVVD
FG10205.1 	GLAGLGSLAENSVRWAAFEDQSQADWIDDIKQASLGNNLKAKRMQQWQIEWEKFCLWIVE
AN2494.1  	GLAGLATMAEDSIRWAAFEDETDSEWLQGIDGK--GNGLKASRLAEWRTAFDEWCAWVVQ
          	**.**.::**:*:**:****::  :*  .:     *.. **.*: :*   ::::* *:*:

MG00770.1 	EYGDKYGEE--------------
NCU04642.1	EYGDEYGGDEA------------
FG10205.1 	EFGDEFGDEPKEAAETKEKANDA
AN2494.1  	EFGAESQI---------------
          	*:* :
```
